# Prediction of posttraumatic stress in fathers of children with chronic diseases or unintentional injuries: a six-months follow-up study

**DOI:** 10.1186/1753-2000-1-16

**Published:** 2007-12-17

**Authors:** Karin Ribi, Margarete E Vollrath, Felix H Sennhauser, Hanspeter E Gnehm, Markus A Landolt

**Affiliations:** 1 International Breast Cancer Study Group, Coordinating Center, Effingerstr. 40, 3008 Bern, Switzerland; 2 Division of Mental Health, Norwegian Institute of Public Health, 4304 Oslo, Norway; 3 Psychological Institute, University of Oslo, 0317 Oslo, Norway; 4 University Children's Hospital, Steinwiesstr. 75, 8032 Zurich, Switzerland; 5 Department of Pediatrics, Cantonal Hospital Aarau, Aarau, Switzerland

## Abstract

**Background:**

While fathers were neglected for a long time in research investigating families of pediatric patients, there are now a few studies available on fathers' posttraumatic stress symptoms (PTSS) and posttraumatic stress disorder (PTSD). However, little is known about the course of PTSS and PTSD in fathers of pediatric patients. The present study aimed to compare the prevalence and course of PTSS and PTSD in fathers of children with different chronic and acute conditions and to identify factors that contribute to fathers' PTSS.

**Methods:**

Sixty-nine fathers of children newly diagnosed with either cancer, type I diabetes mellitus, or epilepsy and 70 fathers of children suffering from an unintentional injury completed questionnaires at 4–6 weeks (Time 1) and six months (Time 2) after diagnosis or injury.

**Results:**

Noticeable PTSD rates were found in fathers of children with a chronic disease (26% at Time 1 and 21% at Time 2, respectively). These rates were significantly higher than rates found in fathers of children with unintentional injuries (12% at Time 1 and 6% at Time 2, respectively). Within six months after the child's diagnosis or accident a decrease in severity of PTSS was observed in both groups. Significant predictors of PTSS at Time 2 were the father's initial level of PTSS, the child's medical condition (injuries vs. chronic diseases) and functional status, the father's use of dysfunctional coping strategies, and father's level of neuroticism.

**Conclusion:**

Our findings suggest that fathers with initially high PTSS levels are at greater risk to experience PTSS at follow-up, particularly fathers of children with a chronic disease. Sensitizing health care professionals to the identification of PTSS symptoms but also to indicators of neuroticism and the use of specific coping strategies early in the treatment course is essential for the planning and implementation of adequate intervention strategies.

## Background

Having one's child being diagnosed with a severe chronic disease or hurt through an unintentional injury is one of the most severe stressors that parents can experience. Parents' often react with posttraumatic stress symptoms (PTSS) or posttraumatic stress disorder (PTSD) [1-7]. PTSS following a traumatic event include persistent frightening thoughts and memories of the ordeal (re-experiencing), avoidance of thinking about the event and feelings of numbness, and increased arousal. PTSD is diagnosed when these symptoms last for more than a month and cause significant functional impairment. In the fourth edition of the Diagnostic and Statistical Manual of Mental Disorders (DSM-IV) [8], learning that one's child has a life-threatening illness qualifies as a traumatic event. Traumatic events can be a single dramatic event (Type I trauma) or repeated traumatic events (Type II trauma) [9].

While fathers were neglected for a long time in research investigating families of pediatric patients, a few more recent studies report on fathers' posttraumatic stress reactions. Elevated levels of PTSS during and after treatment were found in fathers of pediatric cancer survivors [1,2,6,10-12]. In fathers of children with type I diabetes, a significant group met the criteria for full or partial PTSD [13,15]. In a study comparing PTSS and PTSD in fathers of children with different acute and chronic conditions, the highest rates of PTSD were found in fathers of children with newly diagnosed cancer, whereas rates in fathers of children with diabetes and physical injuries were similar [5]. However, in that study the occurrence of PTSS and PTSD was investigated with a cross-sectional design at a relatively early time point in the treatment course (4–6 weeks after diagnosis or injury). Prospective studies of PTSD in fathers examined either fathers of pediatric cancer survivors or fathers of children with newly diagnosed type I diabetes separately [2,15], but no previous study has compared the course of PTSS in fathers of children with different chronic and acute conditions.

Depending on the research focus, interest in factors that predict fathers' adaptation to their child's disease has been selective. Several theoretical models [16-19] have been developed to describe and illustrate predictors of and processes associated with the adaptation of parents to the stress of their child's disease. Most of these models are derived from the stress and coping model [20] and share as a conceptual basis the view that the child's disease is a potential stressor. Cognitive appraisal and coping constitute the central adaptation processes. These processes are influenced by different predictors that can be categorized in illness-related factors (diagnosis, treatment intensity, and others), individual differences (such as socio-demographic variables, personality characteristics), and familial factors (such as social support, family relations).

Regarding illness-related factors, one study reported that the child's functional status and the length of hospitalization were significantly correlated with PTSS levels in fathers of patients with different acute and chronic conditions [5]. In contrast, no or only minimal associations between objective medical parameters (such as intensity of treatment, length of time since diagnosis) and levels of PTSS were found in fathers of pediatric cancer patients or survivors [7,10,11]. Rather, fathers' perceptions of cancer threat (that is, whether the child could still die) and cancer treatment contributed significantly to their PTSS levels.

Few findings exist regarding the role of personality as a predictor of fathers' psychological adjustment. Findings from studies employing the "Big Five" framework of personality domains, which is the gold standard for personality measurement to date, have shown that extraversion, conscientiousness, and agreeableness predict better adjustment to stress, whereas neuroticism predicts poor adjustment [21,22]. However, the Big Five personality domains have never been examined in fathers of pediatric patients. Studies in parents of pediatric cancer survivors that investigated trait anxiety, a measure closely related to neuroticism [23], found that it functioned as a risk factor for the development of PTSS. Whereas one study demonstrated that trait anxiety was a significant predictor of PTSS for both fathers and mothers [11], another found trait anxiety to be a predictor of PTSS in mothers but not in fathers [2]. Among additional psychological predictors investigated, poorer family functioning [24] and satisfaction [10] and lower levels of perceived social support [11,24] were found to be associated with higher levels of PTSS.

To our knowledge no results are available on associations between personality factors other than trait anxiety, subjective appraisal of distress, or coping and PTSS levels in fathers of children with chronic diseases or unintentional injuries. Some limited findings exist regarding psychological symptoms that are associated with coping in fathers of children with different chronic diseases, indicating that fathers relying on strategies such as avoidance coping [25], behavioral disengagement, or venting of emotions [26] report more symptoms.

The first aim of the present study was to compare prevalence and course of PTSD and PTSS between fathers of children with unintentional injuries and fathers of children with a chronic disease during the first six months after the injury or the diagnosis. With respect to prevalence and course of PTSD and PTSS of fathers of unintentionally injured children or fathers of children with a chronic disease we expected the initial levels of posttraumatic stress reactions to be similar in both groups. Over the course of time, however, we expected to find differences between the groups. We hypothesized to see elevated PTSS levels over the first months of the treatment in fathers of children with a chronic disease but declining PTSS levels in fathers of children with unintentional injuries. The second aim of the present study was to examine the role of illness-related factors, personality, family relations, stress appraisal, and coping in predicting fathers' levels of PTSS. We hypothesized, that higher levels in neuroticism, lower levels in extraversion, agreeableness, and conscientiousness, as well as poorer family relations, and the use of dysfunctional coping strategies shortly after the diagnosis or the injury, would predict elevated PTSS levels several months later.

## Methods

### Participants and procedure

A total of 139 fathers of pediatric patients participated. The children and their parents were recruited at four children's hospitals in the German-speaking part of Switzerland. The study was approved by the institutional review-board. Fathers of children who met the following criteria were eligible for the study: 1) a new diagnosis of cancer, type I diabetes mellitus, epilepsy, or the occurrence of an unintentional injury (except severe head trauma), 2) hospitalization for at least 24 hours, 3) child's age between 6.5 and 15 years, 4) fluency in German, and 5) no previous evidence of mental retardation. Because our study also required an interview with the child, children with serious brain injury were excluded. The diagnoses were chosen because they differ in terms of course (chronic vs. acute), and degree of impact on quality of life. All fathers of children consecutively diagnosed were approached.

After giving written informed consent, fathers filled in a set of questionnaires within a period of 4–6 weeks (Time 1) and 6 months (Time 2) after hospital admission. Thus, participants were comparable in terms of time elapsed since the occurrence of the stressor. At Time 1, 173 (84%) from 206 eligible fathers completed questionnaires. Most fathers that did not participate did not live with their child. At Time 2, 22 fathers did not return questionnaires, 11 fathers had withdrawn from the study, and one child had died. The final sample consisted of 139 fathers. Of these, 17%, 26%, and 7% were fathers of children with cancer, diabetes or epilepsy, respectively. Fifty percent were fathers of a child that suffered from an unintentional injury.

There were no significant differences between fathers participating at both Time 1 and Time 2 and fathers participating only at Time 1 with respect to fathers' age and the age or diagnosis of the child. However, fathers of boys declined to participate more often than fathers of girls (χ^2 ^= 4.95; p ≤ .05).

Characteristics of the sample are listed in Table 1. Ninety percent of the fathers were Swiss; 10% originated from other, mostly Mediterranean countries. Thirty-nine children were diagnosed with type 1 diabetes, 23 with cancer, and 9 with epilepsy. Seventy children had an unintentional injury that required hospitalization. Children in the cancer group had a diagnosis of leukemia (N = 9), lymphoma, (N = 5), brain tumors (N = 4), or other solid tumors (N = 5). Children with unintentional injuries had minor head injuries (N = 32), lower-extremity fractures (N = 16), upper-extremity fractures (N = 9), non-extremities fractures (N = 17), internal injuries (N = 8), or burns (N = 16). Twenty-eight of the children suffered from multiple injuries.

**Table 1:** Fathers' and children characteristics

Variable	All		Injuries		Chronic diseases		X^2^	F	p
	N	%	N	%	N	%			
	139	100	70		69				
Marital status							0.72		.39
Married	125	89.9	64	91.4	60	87			
Separated, divorced, remarried	15	10.1	6	8.6	9	13			
Socioeconomic status							5.59		.06
Lower	13	9.4	5	7.1	8	11.6			
Middle	83	59.7	37	52.9	46	66.7			
Upper	43	30.9	28	40	15	21.7			
Education of fathers									
Lower	22	15.8	8	11.4	14	20.3	3.93		.14
Middle	72	51.8	35	50.0	37	53.6			
Higher	44	31.7	27	38.6	17	24.6			
Unknown	1	0.7	-		1	1.4			
Child gender							0.37		.541
Female	60	43.2	32	45.7	28	40.6			
Male	79	56.8	38	54.3	41	59.4			
Child age									
Mean	9.98		9.96		10.19			-0.58	.565
SD	2.38		2.41		2.39				
Days of hospitalization at Time 1									
Mean	11.12		8.53		13.75			-3.71	.000
SD	8.68		7.95		8.65				
Days of hospitalization at Time 2									
Mean	17.71		10.89		24.24			-3.24	.002
SD	25.08		15.56		30.32				

Fathers' socioeconomic status was calculated by means of a score reflecting paternal occupation and maternal education (range 2–12 points) using a measure that has been shown to be a reliable and valid indicator of socioeconomic status in our community [27]. Three social classes were defined including lower class (scores 2–5), middle class (scores 6–8), and upper class (scores 9–12). According to the Swiss education system, a scale of six categories assessed the level of education. Three education levels were defined: lower (categories 1–3), middle (category 4), and higher (categories 5–6) education. Between fathers of children with chronic diseases and fathers of children with injuries there were no differences with regard to father's marital and socioeconomic status, father's education, child's gender, and child's age. Children with a chronic disease had longer hospital stays than children with unintentional injuries at both Time 1 and Time 2.

### Measures

#### PTSS and PTSD

Posttraumatic stress reactions of fathers were assessed using the Posttraumatic Diagnostic Scale (PDS) [28]. This self-report measure of PTSD provides both a diagnosis according to DSM-IV criteria and a measure of PTSD symptom severity. It comprises 17 symptoms of PTSD that are rated on a 4-point Likert scale ranging from not at all (0) to very much (3). The questionnaire also includes one item that assesses the duration of the symptoms using the categories 'less than 1 month', 'one to three months' and 'more than three months'. In addition, nine items assess whether the reaction to the trauma caused impaired functioning in different domains of life (yes/no format). The PDS has demonstrated high internal consistency (α = .92) and good test-retest reliability (α = .74) in its original English version [28]. The agreement between the PTSD diagnosis and the Structured Clinical Interview for DSM-III-R SCID-PTSD module is 82%, the sensitivity of the PDS is .89 and the specificity is .75. The scale is widely used for screening and assessing PTSD in clinical and research settings. The present study used the German version of the PDS [29]. The concurrent validity of PDS symptom severity scores has been supported by high correlations with other measures of psychopathology [29]. In the present study, the internal consistency reached α = .86 (Time 1) and α = .83 (Time 2).

Fathers' posttraumatic stress symptom severity was calculated by summing the 17 PTSD symptoms. In accordance with the criteria of DSM-IV, fathers received a diagnosis of PTSD if they reported the presence of at least one re-experiencing symptom, three avoidance symptoms, and two arousal symptoms. Presence of a symptom corresponds to a rating of 1 or higher on the Likert scale. These symptoms had to occur for at least one month, and had to cause impairment in at least one domain of life [28].

#### Stress appraisal

Stress appraisal was assessed by two single items (appraisal of threat and appraisal of distress). These two items were derived from an appraisal scale that comprises seven different aspects of appraisal. The scale was previously validated in pediatric patients [30]. In the questionnaire, a brief introduction explained the context of each item. The item for threat appraisal referred to perception of dangerousness of the child's disease or injury, while the item for distress appraisal referred to father's subjective distress in reaction to the experience of having a sick or injured child. The answer format consisted of a three-point Likert scale (0–2) with different verbal descriptors for each level. A factor analysis of the seven original appraisal items extracted three factors with appraisal of threat and appraisal of distress loading on the same factor (explained variance: 30.8%). The two items were combined to a single variable, *stress appraisal *ranging from 0–2. Correlations between the two items were r = .42 (p < = .0005) at Time 1 and r = .43 (p < = .0005) at Time 2.

#### Personality

The German version [31] of the NEO-Five Factor Inventory (NEO FFI), a short version of the NEO-PI-R [32], was used to assess fathers' personality. Assuming that personality is a stable construct, the NEO FFI was included in the set of questionnaires to be completed at Time 2 only. The NEO FFI contains 60 items (some of them are reverse coded) reporting on the Big Five personality factors of neuroticism, extraversion, openness for experience, agreeableness, and conscientiousness, with 12 items reporting on each factor. The items are rated on a 5-point scale ranging from strongly disagree (1) to strongly agree (5). In the present study reliability coefficients were .81 for neuroticism, .79 for extraversion, .62 for openness for experience, .62 for agreeableness, and .85 for conscientiousness. The correlations between the NEO FFI scales varied between *r *= .06 (openness for experience with conscientiousness) and *r *= -.50 (neuroticism with extraversion).

#### Medical variables

Two medical variables were included in this study: the child's *medical condition *and *functional status*. Medical condition is a dichotomous variable coded as 0 and 1 indicating injury or chronic disease, respectively. Functional status included two items, one describing the degree to which the child's physical functioning was impaired and the other describing the degree to which child's daily activities were impaired. The child's physician rated these items on 3-point and 5-point Likert type scales with varying verbal anchors. This measure of functional status has been used in prior studies and has proven to be valid [13,33]. Because the two items inter-correlated with Spearman *r *= .68 (p ≤ .0005) at Time 1 and r = .71 (p ≤ .0005) at Time 2, they were standardized and combined to one variable, *functional status *ranging from 0–6.

#### Family relations

Quality of family relations was measured by the German version [34] of the Family Relationship inventory (FRI) that assesses the three relationship subscales *cohesion*, *conflict*, and *expressiveness *of the Family Environment Scale [35]. Each scale is composed of nine items that are scored in a true-false format (*0 *or *1*). Satisfactory levels of reliability [35,36] and support of the construct validity of the FRI have been reported [37,38]. We used the FRI overall index summarizing the three subscales, whereby items of the conflict scale are reverse scored (higher scores mean better family relations, maximum score is 27). Cronbach's alphas of the FRI total scores were 0.78 and 0.80 at Time 1 and Time 2, respectively.

#### Coping

Coping strategies were measured using the Brief COPE [39], an abbreviated version of the COPE Inventory [40]. The Brief COPE comprises 14 strategies that people use to deal with stressful situations. These strategies are labeled active coping, planning, positive reframing, acceptance, humor, self-distraction, denial, substance use, use of emotional support, use of instrumental support, behavioral disengagement, venting, religion, and self-blame. Each strategy is assessed by two items that are rated on a 4-point scale (not at all (0), a little bit (1), a medium amount (2), a lot (3)). To reduce the number of strategies for this analysis, an iterative process was conducted to create second-order factors from the scales as suggested by the authors of the Brief COPE [40]. In a first step, coping strategies with low item-total correlations were excluded. In a second step, the remaining scales were factor analyzed. Strategies making up single factors or loading on several factors were excluded. In a third step, the remaining eight strategies were factor analyzed again, and two factors were extracted. The two factors, labeled 'functional coping' and 'dysfunctional coping,' accounted for 33.9% and 21.6% of the explained variance. Functional coping consisted of the strategies active coping, planning, use of emotional support, and use of instrumental support. Dysfunctional coping comprised the strategies denial, substance use, behavioral disengagement, and self-blame. Scales were computed by summing the four strategies. Reliability coefficients were α = .78 (Time 1) and α = .84 (Time 2) for functional coping and α = .57 (Time 1) and α = .51 (Time 2) for dysfunctional coping.

### Statistical analyses

For bivariate correlations Pearson coefficients were used. Paternal PTSS scores were analyzed using multivariate analysis of variance with time as a within-person and medical condition as a between-person factor. For comparisons of paternal PTSS scores among diagnostic subgroups ANOVA was used with Bonferroni's post-hoc tests. Hierarchical multiple regression analyses were performed to investigate predictors of father's PTSS score at Time 2. Except for the personality variables that were assessed at Time 2, only Time 1 variables were entered into the regression analysis. To control for initial PTSS symptom level, PTSS symptoms scores measured at Time 1 were entered in the regression analysis first. In the second block we entered potential stressors (child's medical condition and functional status). In the third block we included the dispositional variables (personality variables). In the fourth block, all variables supposed to depend on the situation (family relations, stress appraisal, and coping) were entered. The variables of blocks three and four were entered stepwise.

## Results

### Prevalence of PTSD and course of PTSS over time

Twenty-six percent (N = 18) of fathers of children with a chronic disease at Time 1 and 21% (N = 14) at Time 2 met all DSM-IV diagnostic criteria for PTSD. In contrast, 12% (N = 8) of fathers with an injured child met PTSD criteria at Time 1, with a decrease to 6% (N = 4) at Time 2. Differences in the PTSS total score, symptoms of re-experiencing, hyperarousal, or avoidance between the two groups were significant at both time points (Table 2).

**Table 2:** PTSD and PTSS for fathers of children with injuries (N = 70) or chronic diseases (N = 69)

	Injuries	Chronic diseases	X^2^	t	p
Time 1					
% PTSD	12	26	4.34		< .05
PTSS mean total score (SD)	6.77 (6.09)	11.04 (7.36)		-3.72	< .001
Mean re-experiencing score (SD)	3.58 (2.87)	5.13 (3.19)		-3.01	.003
Mean avoidance score (SD)	1.63 (2.17)	3.16 (3.41)		-3.07	.003
Mean hyperarousal score	1.75 (2.25)	3.36 (2.61)		-3.90	< .001

Time 2					
% PTSD	6	22	6.58		< .01
PTSS mean total score (SD)	2.92 (4.61)	6.01 (6.16)		-3.35	.001
Mean re-experiencing score (SD)	1.42 (1.87)	2.83 (2.60)		-3.63	< .001
Mean avoidance score (SD)	0.67 (1.71)	1.76 (2.32)		-3.14	.002
Mean hyperarousal score (SD)	0.84 (1.67)	1.51 (1.94)		-2.19	.03

*Note*. PTSD = Posttraumatic stress disease; PTSS = Posttraumatic stress symptoms; t = independent-sample t tests

PTSS severity varied significantly according to medical condition, with higher scores in fathers of a child with a chronic disease, and it changed (decreased) significantly over time, as shown by a repeated measure analysis of variance (effect of medical condition: F_(1,136) _= 20.4; p ≤ .0005; effect of time: F_(1,136) _= 112.7; p ≤ .0005). However, the course of PTSS over time did not vary with diagnosis (interactive effect of time by medical condition: F_(1,136) _= 0.10; p = .919), which indicates a decrease in symptom severity in both groups over time. Subgroup comparisons (injuries, cancer, and non-cancer) of paternal PTSS scores revealed significant differences between groups at Time 1 (F_(2,136) _= 12.1; p ≤ .0005), and Time 2 (F_(2,135) _= 14.7; p ≤ .0005) with Bonferroni's post hoc tests showing that all three groups differed significantly at both time points.

### Correlations between predictor variables and PTSS scores

Correlations between predictor variables measured at Time 1 (except for the personality variables that were measured at Time 2) and fathers' PTSS scores at both time points are presented in Table 3. Significant correlations were found between child's medical condition and functional status and fathers' PTSS scores. Of the five personality dimensions, neuroticism was significantly associated with higher PTSS scores (at Time 1 and Time 2) while extraversion was significantly associated with lower PTSS scores at Time 2. In addition, fathers' stress appraisal and coping behavior were significantly related to PTSS scores at both times. Fathers who appraised their stress as higher and fathers using functional and dysfunctional coping strategies more frequently showed higher PTSS levels. No significant associations were found between family relations and fathers' PTSD scores either at Time 1 or at Time 2.

**Table 3:** Correlations between Time 1 psychosocial variables and father's PTSS scores at Time 1 and Time 2

Variables	PTSS Time 1	PTSS Time 2
	r	r
Child's medical condition (0 = injury; 1 = chronic disease)	.32**	.33*
Child's functional status	.31***	.42***
Neuroticism	.23**	.33***
Extraversion	.16	-.33***
Openness for experiences	.02	-.12
Agreeableness	.04	-.09
Conscientiousness	.12	-.06
Family relationship index	.02	-.16
Stress appraisal	.56***	.41***
Functional coping	.51***	.38***
Dysfunctional coping	.50***	.50***

*Note*. PTSS = Posttraumatic stress symptoms

* *p *= 0.05; ** *p *= 0.01; * **p = 0.001

### Prediction of PTSS

Results of the regression analysis are shown in Table 4. A total of 52% of the variance in paternal PTSS at Time 2 was explained by initial level of PTSS, child's functional status, dysfunctional coping, child's medical condition, and neuroticism. Fathers experiencing higher levels of PTSS at Time 1 and fathers of a child with a chronic disease were at higher risk for subsequent PTSS. Higher levels of PTSS at Time 2 were also reported by fathers of children who suffered from higher functional impairment at Time 1. In addition, a higher level of neuroticism and the use of dysfunctional coping strategies were significantly associated with higher PTSS levels.

**Table 4:** Final model of stepwise regression analyses predicting paternal PTSS scores at Time 2

Predictors	*B*	*SEB*	*Beta*	*ΔR*^2^	*p*
Step 1					
PTSS Time 1	0.64	0.07	.63		.000
				.40	.000
Step 2					
Child's medical condition (0 = Injury 1 = Chronic disease)	0.40	0.20	.15		.041
Child's functional status	0.73	0.24	.22		.003
				.07	.001
Step 3					
Neuroticism	0.50	0.16	.21		.002
				.04	.002
Step 4					
Dysfunctional coping	0.84	0.31	.21		.007
				.03	.007

Final equation	Adjusted R^2 ^= .52; F_(5,113) _= 26.78; p < .0005				

*Note*. PTSS = Posttraumatic stress symptoms

## Discussion

This study is the first to compare the prevalence and course of PTSD and PTSS among fathers of children with different chronic and acute conditions over a six-months period. Fathers of children with a chronic disease showed considerable rates of PTSD at both time points, amounting to 26% at Time 1 and 21% at Time 2. These rates correspond to the rates of 10% to 30% reported across studies of parents of childhood cancer survivors [41]. Furthermore, researchers assessing families of children with cancer for PTSS rather than PTSD reported that at least one parent showed moderate-to-severe PTSS during the child's treatment [7] in nearly 80% of the families. These symptoms were found in a substantial number of families. In 20% of the families of adolescent cancer survivors, at least one parent had current PTSD even years after treatment had ended [6].

In contrast, only 12% percent of fathers with an injured child met PTSD criteria at Time 1, and this proportion decreased to 6% at Time 2. However, these prevalence rates were still considerably higher than PTSD lifetime prevalence found in a representative community-based adult cohort in Switzerland [42]. In that study none of the persons who reported exposure to a potentially traumatic event met all the criteria for PTSD, and only 0.26 % of males met the criteria for subthreshold PTSD. This comparison shows that fathers of children with unintentional injuries or newly diagnosed chronic diseases are clearly at an increased risk to suffer from PTSD.

With regard to course, our study showed that PTSS severity decreased in both groups during the six months after the child's diagnosis or injury. This is in line with findings reported previously from our study [15], where a subgroup of fathers of children with type 1 diabetes was followed over one year. Also there, a decrease in symptom severity over time was observed [15]. There are no other studies with which we can compare our findings, as the only two earlier studies on PTSS in fathers of children with cancer [2,24] were restricted to a single PTSS measurement after the end of treatment.

Among factors that predict the level or course of PTSS in fathers, we identified five that were of importance. Initial level of PTSS symptomatology at Time 1 was the strongest predictor of fathers' PTSS at Time 2. This suggests that an initial PTSS reaction indicates a heightened vulnerability over time. Having a child with a chronic disease (vs. a child exposed to an injury) was a second risk factor for higher PTSS levels at Time 2. This may reflect Type II trauma [9]. Particularly fathers of children with cancer are likely to experience repeated threats by witnessing their child undergoing painful procedures or suffering from side effects of the treatment. Fathers of children with epilepsy or diabetes may also be confronted with recurring threats such as unforeseen seizures or changes in the health status of their child. The third predictor of fathers' PTSS level at Time 2 was the child's functional status at Time 1, suggesting that the child's impairment in physical functioning and daily life activities may represent a source of threat for the father. In contrast, fathers' subjective appraisal of threat and distress did not predict their PTSS level at Time 2. This is at variance with findings from previous studies showing that fathers' subjective appraisals were more important predictors of their PTSS than objective medical variables [2,11]. The fourth predictor of fathers' PTSS levels at Time 2 was fathers' coping behavior at Time 1. Whereas dysfunctional strategies such as denial, substance use, behavioral disengagement, and self-blame contributed to a higher PTSS level at follow-up, functional strategies were not predictive. This is in line with findings from earlier studies that found negative effects of dysfunctional strategies on fathers' well being [43] and no effects for functional strategies [25,26,44,45]. However, conclusions have to be drawn with caution, because the internal consistency of the scale for dysfunctional coping was rather low. Finally, also fathers' personality, notably neuroticism, predicted their levels of PTSS at Time 2. This accords with findings showing that trait anxiety is associated with PTSS after a traumatic event in general [46,47] and after the cancer of one's child in particular [2,11]. No other personality factor was associated with PTSS prospectively.

### Limitations

This study has some limitations that merit mention. PTSD was assessed by a questionnaire with sufficient psychometric properties, but without direct clinical interviews. As there is no complete agreement between these two measurement methods [28] there may be a risk for false positive or false negative cases. Our PTSD prevalence rates have therefore to be considered as an estimation of PTSD rates. Although the predictors included in the multivariate analysis explained a considerable amount of variance, other factors not measured in this study may have an impact on fathers' PTSS. The child's psychological reaction, for example, was not considered, although associations between a father's and his child's emotional condition have been reported [48]. For instance, the severity of paternal PTSS in an early period following a child's accident predicted child's PTSS one year after a road accident [14]. In addition, regression analysis is an exploratory tool. A further limitation concerns the a priori classification of the different diagnoses in acute versus chronic conditions. In particular, the diseases in the chronic category are heterogeneous. For instance, as the potential life-threat is much higher for oncological diseases than for diabetes or epilepsy, different psychological reactions may ensue. Within the group of patients with acute conditions, injury severity varied considerably, ranging from minor (such as concussions) to severe (burn injuries, for example). Some children suffering from burns may experience long-term sequelae that are similar to sequelae that result from chronic disease [49]. Finally, we cannot estimate the extent to which social desirability biased the fathers' responses. In spite of the increasing similarity of gender roles today, fathers still are expected to be strong and supporting rather than to admit their vulnerability.

### Clinical implications

Regardless of these limitations, the present study has two major strengths. First, it evaluates PTSD and PTSS in fathers of pediatric patients not only at a single point in time but over a period of six months after the diagnosis of chronic disease or after the accident. Studies using a longitudinal design are still few in number. Second, two groups of fathers are compared that are assumed to differ with respect to their responses to a potential traumatic event. Taking this into account, our findings suggest several clinical implications. The finding that a substantial proportion of fathers of children with a chronic disease met the criteria for a PTSD diagnosis at both time highlights the importance of identifying fathers at risk for PTSD at an early stage of the treatment.

Health care professionals should be sensitized to the appearance of symptoms related to posttraumatic stress as well as to potential traumatic situations during the child's treatment. It is essential to identify symptoms such as intrusive thoughts, avoidance, and arousal in fathers, as they indicate the need for early intervention. Brief intervention strategies have been shown to result in a significant reduction of intrusive thoughts among fathers of adolescent cancer survivors [50]. Furthermore, it is important to be aware of fathers with elevated levels of neuroticism, as they are at higher risk to develop PTSD. While strategies or interventions to minimize the child's anxiety have become part of comprehensive medical care in pediatric settings, fears and worries of fathers may be less apparent, because fathers are not expected to express their fears and because they are less present at the hospital. Therefore, fathers should be involved in discussions with health care professionals whenever possible, and they should be encouraged to vent their worries. Fathers who use coping strategies such as denial of the situation, behavioral disengagement, and self-blame should be recognized, as they may be at risk for developing PTSS.

## Conclusion

Our findings suggest that fathers with initially high PTSS levels are at greater risk to experience PTSS at a later date, particularly fathers of children with a chronic disease. Sensitizing health care professionals to the identification of PTSS symptoms, but also to indicators of neuroticism and specific coping strategies early in the treatment course is essential to the planning and implementation of adequate intervention strategies.

## Competing interests

The author(s) declare that they have no competing interests.

## Authors' contributions

This work bases on the doctorial dissertation of KR at the University of Zurich, Switzerland. KR conceived the design of this study, performed the data analysis and drafted the manuscript. MEV was KR's doctorial advisor. She designed the study, advised with respect to the analysis and interpretation of data and participated in the drafting and revision of the manuscript. FHS and HEG participated in the design of the study, the acquisition and interpretation of data. MAL designed the study, participated in the collection and analysis of data and revised the manuscript for important intellectual content. All authors read and approved the final manuscript.
